# Mitogenomic phylogeny of the aquatic subterranean Bathynellacea (Crustacea, Malacostraca) and implications for the monophyly of Syncarida

**DOI:** 10.1038/s41598-026-56722-z

**Published:** 2026-06-15

**Authors:** Su-Jung Ji, Ji-Hun Song, Chi-Woo Lee, Gi-Sik Min, Se-Joo Kim

**Affiliations:** 1 Metabolic Regulation Research Center, Korea Research Institute of Bioscience and Biotechnology, Daejeon, 34141 South Korea; 2 Department of Biological Sciences and Bioengineering, Inha University, Incheon, 22212 South Korea; 3 Animal and Plant Research Division, Nakdonggang National Institute of Biological Resources, Sangju, 37242 South Korea

**Keywords:** Bathynellacea, Mitogenome, Syncarida, Polyphyly, Convergent evolution, Subterranean adaptation, Ecology, Ecology, Evolution, Genetics, Zoology

## Abstract

The crustacean order Bathynellacea is a specialized monophyletic lineage restricted to aquatic subterranean environments and comprises approximately 340 extant species from three families. Despite advances in sequencing technologies that have significantly increased the number of sequenced crustacean mitogenomes, no bathynellacean mitogenomes have been reported to date. In this study, we report the first complete mitogenomes for Bathynellacea—*Allobathynella* sp., *Arisubathynella cheongmiensis*, *Hangangbathynella mihoensis* (Parabathynellidae), and *Bathynella* cf. *rufa* (Bathynellidae)—from two families. These mitogenomes, ranging from 14,422 to 16,645 bp in length, are characterized by extensive gene rearrangements, pronounced compositional biases, and accelerated evolutionary rates. Phylogenetic analyses based on malacostracan mitogenomic data strongly support the monophyly of Bathynellacea. However, the exceptionally long branches separating bathynellacean taxa suggest that extensive morphological simplification, driven by parallel adaptations to subterranean environments, may mask the ancestral diagnostic signals required to resolve their deep-level relationships. Most notably, our results indicate that the two extant orders within the superorder Syncarida—Bathynellacea and Anaspidacea—are phylogenetically distant, supporting the polyphyly of Syncarida. This suggests that their shared morphological features (e.g., the absence of a carapace and the loss of the mandibular lacinia mobilis) are likely products of convergent evolution rather than common ancestry. Our study provides mitogenomic resources for Bathynellacea and underscores the necessity of mitogenomic evidence in reassessing the taxonomic status of subterranean crustacean lineages.

**Supplementary Information:**

The online version contains supplementary material available at https://doi.org/10.1038/s41598-026-56722-z.

## Introduction

Bathynellacea Chappuis, 1915 is a crustacean order comprising small (typically 0.5–2 mm) eyeless species that are restricted to aquatic subterranean environments worldwide, except for Antarctica^1–3^. These crustaceans exhibit morphological adaptations suited to their habitats, characterized by a vermiform body shape and reduced appendages^1,3^. Bathynellacea currently includes three families, Bathynellidae, Parabathynellidae, and Leptobathynellidae, encompassing approximately 340 described species across 82 genera^4,5^. However, their true species diversity is estimated to exceed 1000 species when considering their high potential for allopatric speciation because of their limited migration between fragmented aquatic subterranean habitats^5,6^. Compared with other crustaceans, phylogenetic studies on bathynellaceans are relatively recent and limited^3,7^. Most have relied on partial gene sequences, particularly nuclear 18S and 28S rRNA and mitochondrial *COX1* genes, providing only a partial understanding of their evolutionary history at the molecular level.

Mitogenomes, which include features such as gene content and arrangement as well as nucleotide and amino acid composition, provide strong phylogenetic resolution and serve as valuable molecular markers to clarify relationships among diverse metazoan lineages, particularly when phylogenies based on partial gene sequences remain unresolved^8–13^. In fact, across numerous taxa, including insects, mollusks, and crustaceans, mitogenomes have long been used as effective tools for resolving ambiguous phylogenetic relationships^14–16^. Recently, the development of next-generation sequencing technologies has greatly enhanced the efficiency and cost-effectiveness of mitogenomic research, leading to a dramatic increase in available mitogenome data across both animal and plant taxa^17,18^.

The superorder Syncarida Packard, 1885, which includes two extant orders, Bathynellacea and Anaspidacea Calman, 1904, has traditionally been regarded as a monophyletic group based on shared morphological characteristics (e.g., absence of a carapace, elongated segmented body, reduced visual organs associated with subterranean habitats), similar life history traits (e.g., direct development without free-swimming larval stages), and a common association with subterranean habitats in Bathynellacea and some lineages of Anaspidacea^19–22^. However, several studies have questioned the monophyly of Syncarida based on aspects such as their contrasting geographical distributions (Anaspidacea restricted to the Southern Hemisphere vs. Bathynellacea with worldwide distribution), morphological differences (complex antennae, mandibles, maxillae, and foregut in Anaspidacea vs. simplified structures in Bathynellacea), and inconsistent phylogenetic placement in molecular analyses^22–26^. The recent availability of complete mitogenome data for Anaspidacea has provided new opportunities to reassess its phylogenetic position within Malacostraca^27^. However, no mitogenome has been reported for Bathynellacea, and the mitogenomic phylogeny of members of Syncarida remains unresolved.

In this study, we present the first determined mitogenomes from bathynellacean species, representing four genera across two families. To resolve phylogenetic relationships within Bathynellacea, we characterized these mitogenomes and constructed phylogenetic trees incorporating available malacostracan mitogenomes, including anaspidacean data from public databases. Based on the resulting phylogenies, we further address taxonomic ambiguities among syncarid members.

## Results and discussion

### General features of bathynellacean mitogenomes

**Fig. 1:**
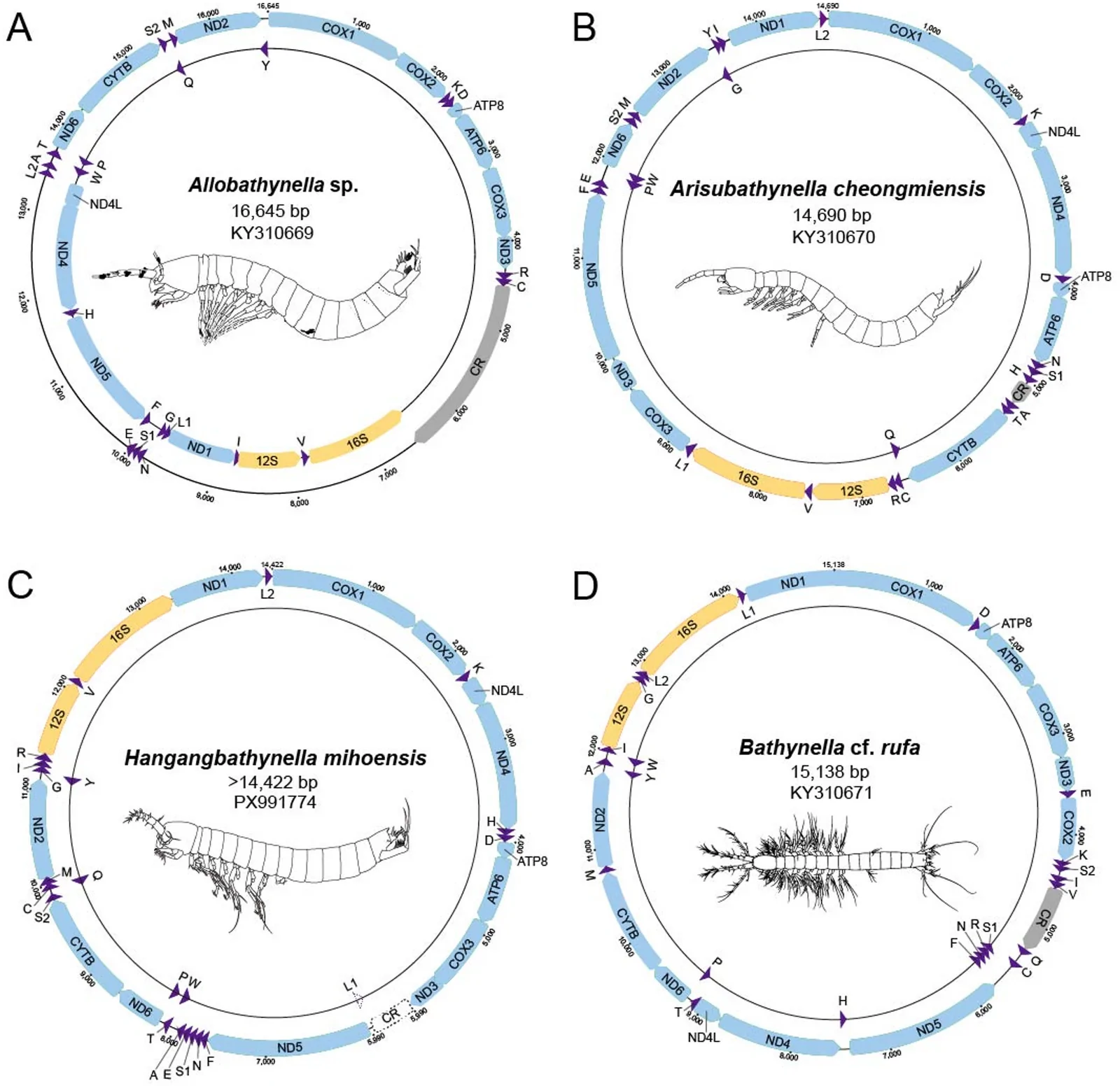
Circular maps of four bathynellacean mitogenomes (**A**–**D**), illustrating genome sizes and gene organizations.

We sequenced four mitogenomes from bathynellacean species, of which three were parabathynellids, and one was bathynellid (Table 1; Fig. 1). This study represents the first report on the mitogenomes of Bathynellacea, comprising three complete mitogenomes and one incomplete mitogenome for *H. mihoensis*, in which a region containing CR could not be recovered. The three complete mitogenomes (*Allobathynella* sp., *Arisubathynella cheongmiensis *Park & Eun, 2012^45^ and *Bathynella* cf. *rufa *Morimoto, 1970^44^ ranged from 14,690 to 16,645 bp in length, with size variation largely attributable to differences in the CR, which varied from 224 to 2064 bp. These mitogenomes contained 13 PCGs, two ribosomal RNA genes, 22 tRNA genes, and a CR. While the mitogenome of *Hangangbathynella mihoensis* Ji & Min, 2021^46^ was incompletely assembled, yielding a sequence length of 14,422 bp and no identifiable CR was recovered, and only 21 tRNAs were annotated due to the failure to detect trnL1.

**Table 1:** Key features of syncarid mitogenomes included in the comparative mitogenomic analyses.

Order	Family	Species	Length (bp)		AT content (%)		AT skew		GC skew		References
			Entire	PCG	Entire	PCG	Entire	PCG	Entire	PCG	
Bathynellacea	Parabathynellidae	*Allobathynella* sp.	16,645	10,972	71.7	72.5	− 0.016	0.003	− 0.062	− 0.037	This study
		*Arisubathynella cheongmiensis*	14,690	10,860	78.9	77.9	− 0.106	− 0.414	0.05	0.025	This study
		*Hangangbathynella mihoensis*	> 14,422^†^	10,945	n/a	79.7	n/a	− 0.133	n/a	0.047	This study
	Bathynellidae	*Bathynella* cf. *rufa*	15,138	10,819	82.2	81.2	− 0.02	− 0.062	0.071	0.067	This study
		Average (± SD)	15,224 (± 993)	10,906 (± 78)	77.6 (± 5.4)	77.8 (± 3.7)	− 0.047 (± 0.051)	− 0.152 (± 0.184)	0.020 (± 0.072)	0.026 (± 0.045)	
Anaspidacea	Anaspidesidae	*Allanaspides helonomus*	15,309	11,142	69	66.4	− 0.082	− 0.197	− 0.307	− 0.036	Höpel et al., 2023
		*Allanaspides hickmani*	15,568*	11,130	70.7	68.3	− 0.086	− 0.197	− 0.302	− 0.031	Höpel et al., 2023
		*Anaspides clarkei*	15,213	11,124	71.8	69.5	− 0.086	− 0.199	− 0.233	− 0.005	Höpel et al., 2023
		*Anaspides swaini*	15,514	11,124	71.8	69.6	− 0.133	− 0.214	− 0.217	0.012	Höpel et al., 2023
		*Anaspides richardsoni*	15,906	11,124	71.2	68.7	− 0.117	− 0.219	− 0.247	− 0.004	Höpel et al., 2023
		*Anaspides jarmani*	17,962	11,130	73.8	69.4	− 0.093	− 0.199	− 0.249	− 0.005	Höpel et al., 2023
		*Anaspides eberhardi*	15,670*	11,124	71.4	68.6	− 0.124	− 0.22	− 0.229	0.007	Höpel et al., 2023
		*Anaspides spinulae*	16,667	11,124	71.8	68.8	− 0.123	− 0.216	− 0.24	− 0.001	Höpel et al., 2023
		*Anaspides driesseni*	16,128	11,124	72.3	69.4	− 0.115	− 0.209	− 0.238	0.002	Höpel et al., 2023
		*Anaspides tasmaniae*	15,475	11,124	72.9	70.8	− 0.123	− 0.214	− 0.25	0.012	Höpel et al., 2023
		*Paranaspides lacustris*	15,779	11,124	69.2	66	− 0.05	− 0.206	− 0.223	0.013	Höpel et al., 2023
		*Paranaspides williamsi*	17,320	11,124	69.6	65.3	− 0.052	− 0.197	− 0.216	− 0.006	Höpel et al., 2023
	Koonungidae	*Micraspides* sp.	16,392	11,130	75.5	72.7	− 0.023	− 0.179	− 0.275	0.006	Höpel et al., 2023
		Average (± SD)	16,070 (± 890)	11,127 (± 5)	71.6 (± 1.8)	68.7 (± 2.0)	− 0.093 (± 0.034)	− 0.205 (± 0.012)	− 0.248 (± 0.030)	− 0.003 (± 0.015)	

^†^Incomplete mitogenome not recovering a control region.

*Mitogenome containing ambiguous nucleotides in a control region.

**Fig. 2:**
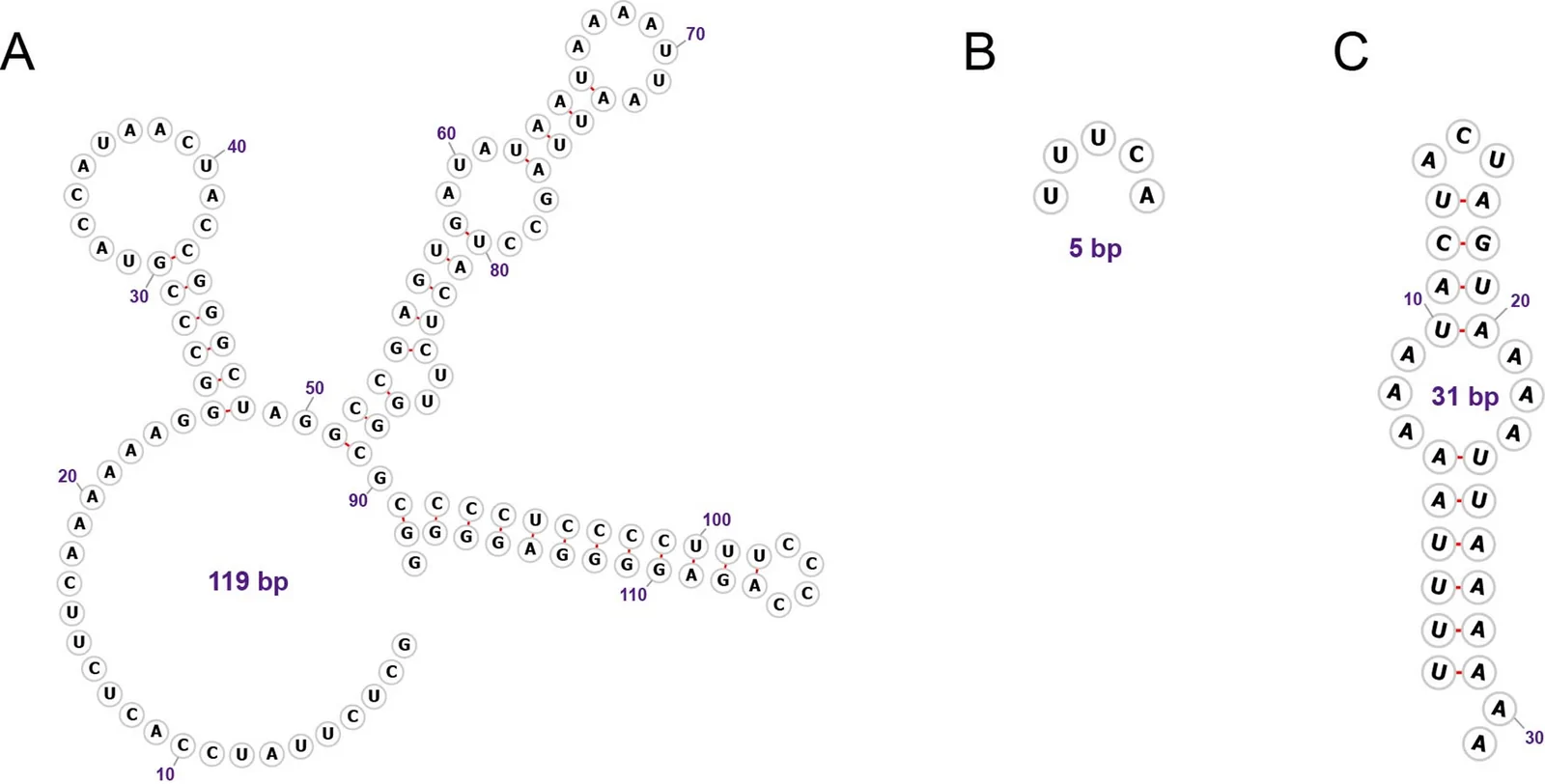
Predicted secondary structure of a repeat unit in the control region of parabathynellid *Allobathynella* sp. (**A**) and *Arisubathynella cheongmiensis* (**B**), and bathynellid *Bathynella* cf. *rufa* (**C**).

Across the four species, the total length of 13 PCGs ranged from 10,819 to 10,972 bp (75.7–81.2% AT content); 12S and 16S rRNA genes ranged from 729 to 769 bp (77.5–84.9% AT content) and 1,196 to 1,253 bp in length (75.7–84.6% AT content), respectively. The tRNAs mostly formed a typical cloverleaf secondary structure, with a few exceptions (Supplementary material Fig. S1). Additionally, tandem repeat motifs were identified within the CR, consisting of 119 bp repeated nine times in *Allobathynella* sp. and 5 bp repeated five times in *Ar. cheongmiensis*, and 31 bp were repeated 21 times in *B*. cf. *rufa* (Supplementary material Table S2). The secondary structures of these regions exhibited stem-loop formations in *Allobathynella* sp. and *B*. cf. *rufa* and linear repetitive patterns in *Ar. cheongmiensis* (Fig. 2). Because CR is essential for initiating mitochondrial DNA replication and transcription, these repetitive motifs are presumed to play a role in the binding of regulatory proteins, recognition of replication origins, and modulation of mutation rates or replication efficiency^28–30^.

Notably, genetic divergences among the three parabathynellid genera ranged from 20.1–25.1% for the *COX1* gene to 39.9–54.9% for the *ATP8* gene—comparable to the divergence between the two anaspidacean families, Anaspidesidae and Koonungidae, which ranged from 19.0–51.7%, across all PCGs^27^. These findings suggest that bathynellaceans have undergone markedly different mitogenomic evolutionary histories, particularly in terms of nucleotide substitution patterns, compared with other syncarid members. The accelerated mitogenomic divergence observed in Bathynellacea aligns with patterns reported in other subterranean arthropods, such as the fossorial crayfish *Engaeus* and troglomorphic cockroaches Nocticolidae^31,32^. These elevated substitution rates suggest that the transition from terrestrial surface to subterranean habitats may impose common selective pressures on mitogenomic architecture. Beyond habitat-driven factors, the distinct evolutionary trajectory of the Bathynellacea mitogenome may also reflect a combination of specialized environmental constraints and an isolated life history, which together could contribute to the unique genomic patterns observed in this lineage.

### Phylogenetic status of Bathynellacea and intragroup relationship

**Fig. 3:**
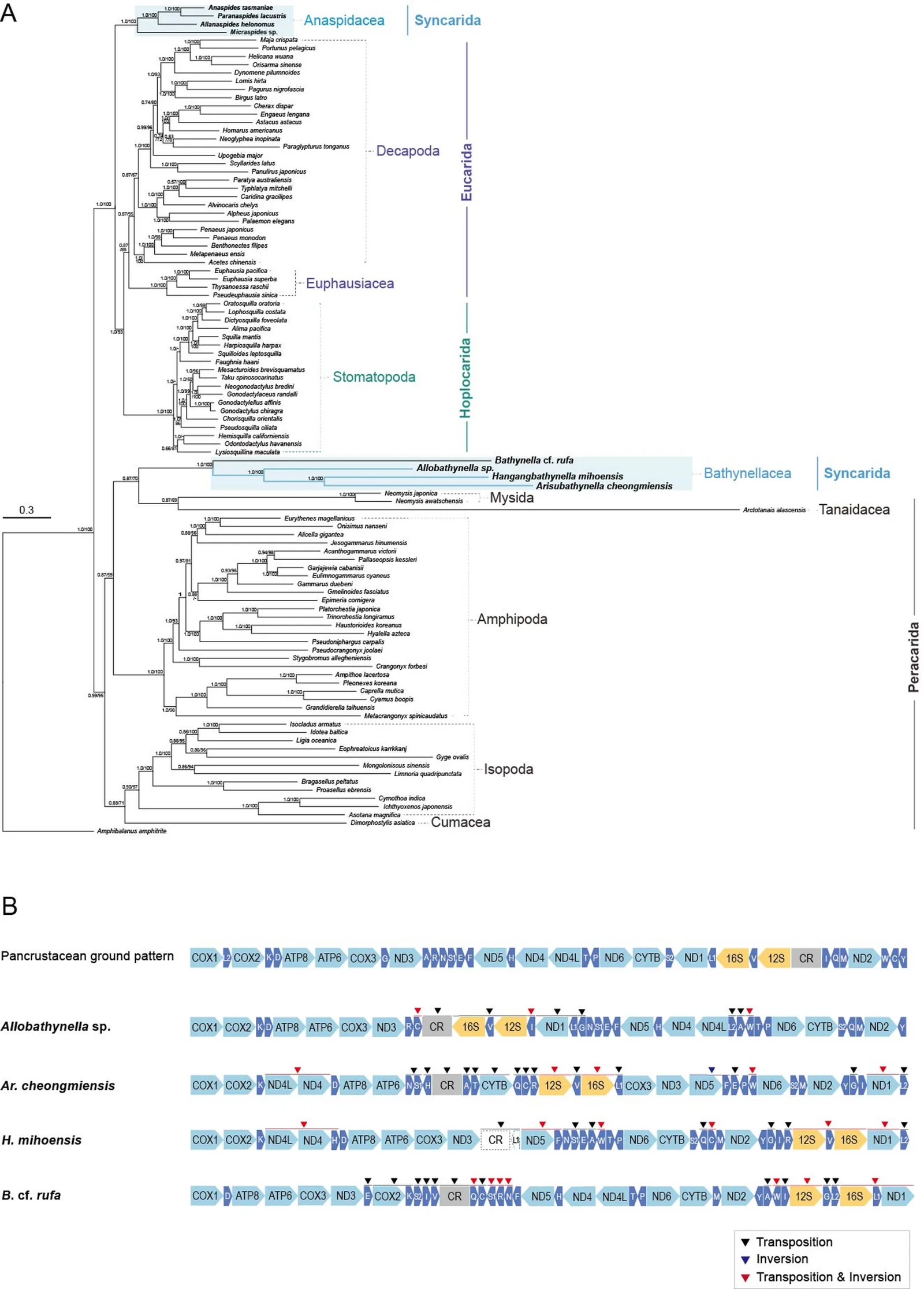
Analysis based on protein-coding genes from malacostracan mitogenomes. Bayesian inference (BI) phylogenetic tree (**A**), with posterior probabilities (left) and maximum likelihood (ML) bootstrap values (right) shown at each node, and mitochondrial gene organization of Bathynellacea mitogenomes (**B**). Hyphens denote bootstrap values < 50.

To clarify the phylogenetic position of the order Bathynellacea and the relationships among its members, phylogenetic trees were reconstructed using BI and ML methods based on a concatenated alignment of the 13 PCGs from the mitogenomes of four bathynellacean species and 96 additional malacostracans (Supplementary material Table S1). The alignment was 12,318 bp long and contained 1483 identical sites. Both BI and ML analyses produced largely congruent topologies, with no differences in relationships among major lineages (Fig. 3A). The trees strongly supported the monophyly of the Bathynellacea (posterior probability = 1.0; bootstrap value = 100%). Within the Bathynellacea, the family Parabathynellidae, which includes multiple species, forms a well-supported monophyletic lineage with full support. While additional mitogenomes are required to fully evaluate the phylogenetic status of the family Bathynellidae.

In terms of gene organization, most malacostracan crustaceans—including other syncarids such as anaspidaceans—exhibited a well-conserved arrangement of PCGs and rRNAs that largely reflected the pancrustacean ground pattern, although tRNAs are occasionally rearranged among closely related species (e.g., trnL1, trnW, and trnL2 in Anaspidacea)^27^. In contrast, bathynellaceans showed extensive gene rearrangements involving not only tRNAs, but also PCGs and rRNAs, diverging markedly from the pancrustacean ground pattern (Fig. 3B; Supplementary material Fig. S2). These findings indirectly suggest that bathynellacean mitogenomes have undergone distinct evolutionary trajectories in gene rearrangement, possibly resulting from long-term divergence from a common ancestor, or from rapid evolutionary processes associated with adaptation to isolated subterranean habitats^31–33^. However, further evidence is needed to confirm this interpretation.

### Distinctive mitogenomic characteristics observed among parabathynellid genera

Based on previous morphological and molecular phylogenetic studies, parabathynellid species have been consistently recognized as a monophyletic group, distinguished from bathynellids based on characteristics, such as the absence or reduction of the first pleopod, posteriorly directed and reduced antennae, and a mandibular palp reduced to a single seta^1,4,34^. In the phylogenetic trees constructed in this study, the three parabathynellid genera formed a single clade that was recovered as a sister group to the bathynellid genus (Fig. 3A). However, the branches separating these genera were exceptionally long, comparable to or exceeding those typically observed among distinct malacostracan orders, indicating substantial genetic divergence within parabathynellids. This pronounced divergence may have contributed to the topological instability of this taxon. This pattern suggests that the long branches in bathynellaceans might reflect the combined effects of adaptive convergence under specialized ecological constraints and restricted dispersal capabilities inherent to their stygobitic nature, along with potential artifacts arising from the limited sampling of intermediate taxa^35,36^.

Based on mitochondrial gene organization, tRNA genes in parabathynellids were frequently transposed even among closely related species; more notably, transpositions of PCGs, which are rare within families, were also observed (Fig. 3B; Supplementary material Fig. S2). The most conspicuous features were found in *Hangangbathynella* and *Arisubathynella*, where all PCG and rRNA genes were located on heavy strands. This contrasts with *Allobathynella*, which exhibits a split arrangement with genes distributed on both the heavy and light strands relative to the CR. Furthermore, *Allobathynella* resembles the ancestral gene arrangement, except for a block on the light strand (12S–16S–ND1) that appears to have undergone approximately two transposition events, resulting in a rearranged ND1–12S–16S configuration, compared with that in the pancrustacean ground pattern. In contrast, other parabathynellid genera exhibit highly derived gene orders resulting from multiple transpositions, inversions, and inverse transpositions across several mitogenomic regions, including the 12S–16S–ND1 block. These findings suggest that *Allobathynella* possesses the most ancestral mitogenomic architecture among the three genera, and that at least two major independent gene rearrangement events occurred within parabathynellid members.

**Fig. 4:**
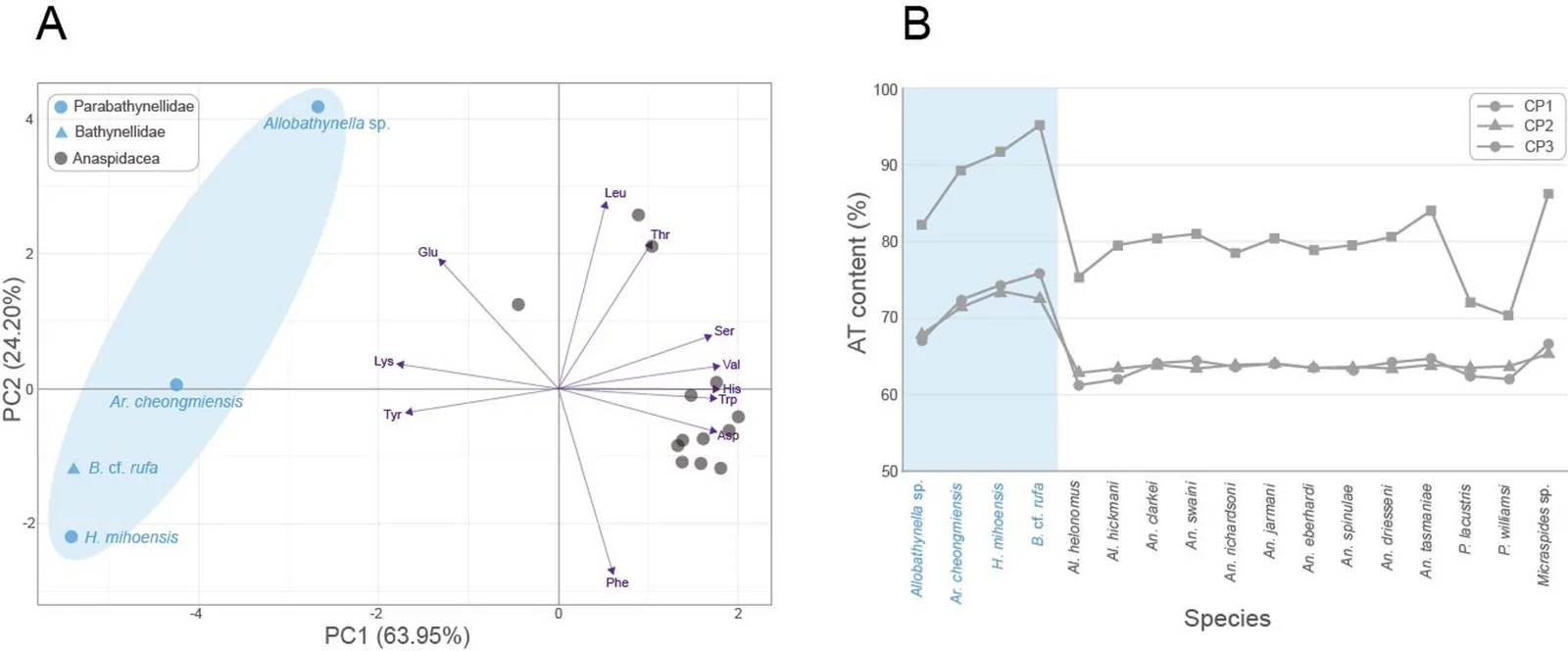
Comparative analysis of syncarid mitogenomes. Principal component analysis biplots illustrating the compositions of amino acids that are normally distributed (*p* > 0.05) (**A**) and AT content at each codon position (**B**) in 13 mitochondrial protein-coding genes.

**Fig. 5:**
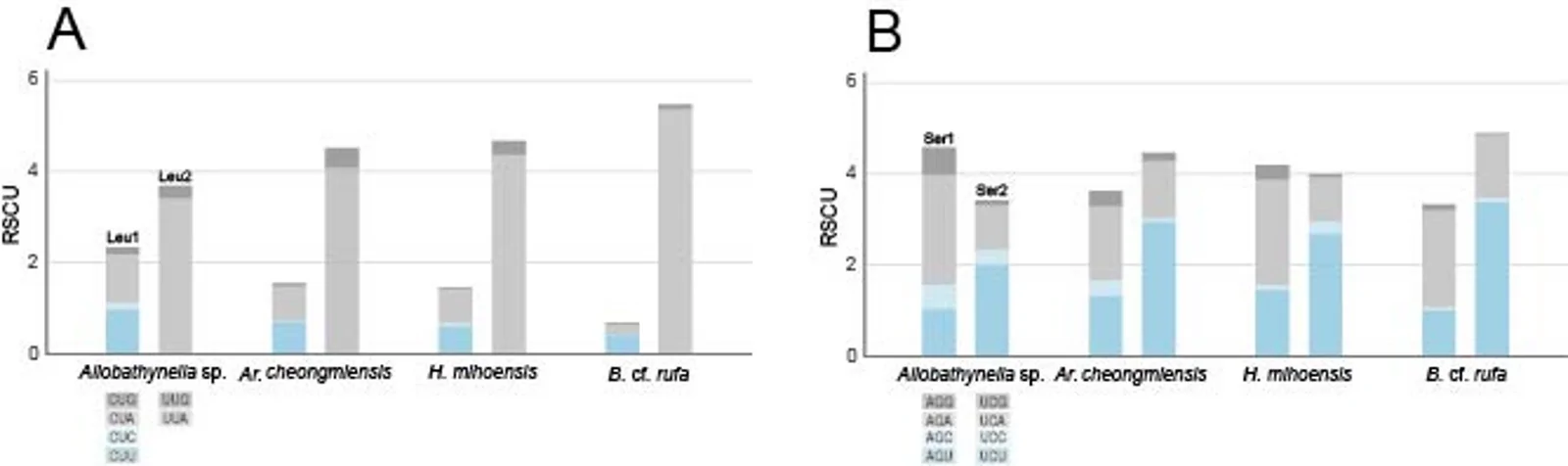
Proportions of leucine and serine codons encoded by two different mitochondrial genetic codes in protein-coding genes of four bathynellacean mitogenomes.

Regarding nucleotide composition in PCGs between the parabathynellids, *Allobathynella* exhibited a notably lower AT content compared to *Arisubathynella* and *Hangangbathynella* (72.5% vs 77.9–79.7%; Table 1). Additionally, *Allobathynella* showed a positive AT skew and negative GC skew, indicating an AC compositional bias, whereas *Arisubathynella* and *Hangangbathynella* displayed a negative AT skew and positive GC skew, suggesting a TG compositional bias (Table 1**)**. To investigate the patterns in amino acid composition, PCA was conducted on the frequency data of amino acids normally distributed across the PCGs (Fig. 4A; Supplementary material Table S3). PC1 and PC2 accounted for 88.15% of the total variance. *Allobathynella* was clearly separated from the other parabathynellids and bathynellid, being associated with moderately negative PC1 and strongly positive PC2 values, in contrast to the others, which showed negative values for both components. Moreover, the codon proportions of two amino acids, leucine (Leu1 and Leu2) and serine (Ser1 and Ser2), each encoded by two distinct mitochondrial genetic codes, differed markedly between *Allobathynella* and the other genera (38.8% vs 23.7–25.5% for Leu1 and 57.3% vs 44.7–51.2% for Ser1; Fig. 5). For alanine as well, *Allobathynella* exhibited distinct codon usage patterns compared to the other genera (Supplementary material Table S4). These pronounced differences in nucleotide composition, amino acid profiles, and codon usage suggest that *Allobathynella* underwent distinct selective pressures during mitogenomic evolution relative to the other two genera. Similar cases of revised relationships based on mitogenomic evidence have been reported in other crustacean groups (e.g., Decapoda thalassinida), even though traditional taxonomic and phylogenetic studies have previously regarded them as monophyletic lineages sharing a common ancestor^37,38^.

### Phylogenetic complexity within Syncarida revealed by mitophylogenetic analysis

Based on the mitogenomic phylogenetic trees, malacostracan taxa formed a well-supported monophyletic group in both BI and ML analyses, with most constituent superorders forming robust monophyletic lineages (Fig. 3A). Despite their stability, Anaspidacea and Bathynellacea were placed in two phylogenetically distant clades (Syncarida I and Syncarida II in Fig. 3A; Supplementary material Fig. S3). This separation suggests that the traditionally defined superorder Syncarida is not monophyletic. Specifically, Bathynellacea was closely related to Peracarida, whereas Anaspidacea was associated with Eucarida and Hoplocarida, which may represent one of several possible evolutionary scenarios within Malacostraca.

To better understand the phylogenetic relationships within Syncarida, we examined additional mitogenomic features, including nucleotide and amino acid compositions (Fig.4; Table 1). One notable finding was the difference in the average total length of the 13 PCGs, which were shorter in Bathynellacea (10,906 bp) than in Anaspidacea (11,127 bp), primarily because of the 27-amino acid difference in the *ND5* gene. In addition, no shared gene rearrangements were identified between Bathynellacea and Anaspidacea based on comparisons with previously reported anaspidacean mitogenomes (Fig. 3B)^27^. Nucleotide composition also differed markedly, with Bathynellacea (77.8%) showing a higher AT content than Anaspidacea (68.7%), a pattern consistently reflected across all codon positions. Both groups showed no significant AT skew in terms of nucleotide compositional bias; however, Anaspidacea displayed more strongly negative GC skew than those in Bathynellacea at the whole-mitogenome level. Furthermore, PCA of amino acid compositions revealed distinct clustering, in that all bathynellacean species were associated with negative PC1 values, whereas all anaspidacean species, except *Micraspides* sp., were associated with positive PC1 values. These unique mitogenomic architectures, characterized by extensive gene rearrangements and divergent compositions, provide a suite of discrete evolutionary characters that distinguish these lineages. The pronounced discordance between traditional morphological classifications^19–22^ and the mitogenomic divergences revealed in this study suggests that their anatomical similarities—particularly reductive features relative to the generalized eumalacostracan body plan, observed in Bathynellacea and certain subterranean lineages of Anaspidacea, including the loss of the carapace, absence or reduction of compound eyes, simplification of mandibular structures, and reduction of maxillipeds and pleopods (Table 2)—likely represent instances of convergent evolution. These shared traits were likely driven by parallel adaptations in both lineages to the selective pressures of the dark and interstitial spaces of subterranean environments, rather than reflecting a recent common ancestry. Furthermore, this extensive morphological simplification, characterized by the loss or reduction of complex ancestral traits to maximize energy efficiency, masks the diagnostic signals required to verify their independent origins, thereby limiting the utility of morphological data for reconstructing their true evolutionary histories. This explains why the close affinities recovered in our analysis, specifically between Bathynellacea and Peracarida, as well as between Anaspidacea and the Eucarida-Hoplocarida clade, have remained morphologically obscured and unrecognized under traditional classification systems. Thus, our mitogenomic results provide critical evidence that the similarities between these two syncarid orders represent adaptive convergence driven by shared environmental constraints rather than common ancestry. It is consistent with the non-monophyletic status of Syncarida recovered in recent large-scale genomic analyses (Fig. 3A)^25,36^, and is further supported by the pronounced ecological, biogeographic, and morphological divergences between Bathynellacea and Anaspidacea (Table 2).

**Table 2:** Key diagnostic and biological attributes of the two extant syncarid orders, Bathynellacea and Anaspidacea.

Feature	Feature	Bathynellacea Chappuis, 1915	Anaspidacea Calman, 1904
Biogeography	Distribution	Worldwide (Cosmopolitan)	Southern hemisphere (Australia, New Zealand, Chile, and Argentina)
Ecology	Habitat	Exclusively subterranean water	Subterranean and surface water
Development	Free-living larval stage	Absent	Absent
Morphology	Carapace	Absent	Absent
	Eye	Absent	Present (stalked and sessile) or absent
	Antennular articles (peduncle + major flagellum)	6–15	23–95
	Lacinia mobilis in mandible	Absent	Absent
	Maxillular endites	2	2
	Maxillary endites	3–4	4
	Maxilliped pairs	0	1
	Fused/free thoracic somites	0/8	1/7
	Pleonal somites (free)	5	6
	Pleopod pairs	0–1	2–6
	Male copulatory organ	Derived from thoracopod VIII	Derived from pleopods I and II
	Uropodal protopod articles	1	1

Recent nuclear phylogenomic studies of Pancrustacea support the paraphyly of the two syncarid orders; however, the specific placement of malacostracan constituent orders remains inconsistent across different datasets^25,36^. In contrast, our mitochondrial dataset indicates a polyphyletic relationship between these two orders (Fig. 3A). These incongruences likely reflect the distinct evolutionary signals inherent in mitochondrial versus nuclear genomes^39,40^. While nuclear phylogenomics integrates signals from thousands of orthologous loci to provide a consensus of ancestral relationships, the mitochondrial genome is inherited as a single evolutionary trajectory and a non-recombining unit with a significantly higher substitution rate. In Bathynellacea, which exhibit extreme mitogenomic divergence, this unified mitogenomic signal may be more susceptible to phylogenetic artifacts, such as long-branch attraction, than large-scale nuclear data^41,42^. Consequently, the placement of Bathynellacea in our analysis provides an independent evolutionary perspective that, when integrated with nuclear genomic data, underscores the complex and potentially multiple origins of syncarid-like lineages within Malacostraca.

Our mitogenome-based analyses provided important insights into the evolutionary history of Bathynellacea and its relationship with other syncarid members. Pronounced differences in mitogenomic features, particularly in *Allobathynella*, indicate that this genus has undergone distinct selective pressures compared with other parabathynellid lineages. These molecular distinctions, along with the presence of exceptionally long phylogenetic branches, may reflect convergent evolutionary responses to similar subterranean environments or may result from the limited sampling of intermediate taxa. Importantly, our results show that the two syncarid orders, Bathynellacea and Anaspidacea, do not form a monophyletic group, indicating that they do not share a recent common ancestor, despite sharing ecological and morphological traits. Consequently, to better understand the migration patterns of parabathynellids across isolated habitats and clarify the relationships among aquatic subterranean crustacean lineages, our findings highlight the need for further research incorporating additional taxa and expanded comparative genomic analyses.

## Materials and methods

### Sample collection and species identification

**Fig. 6:**
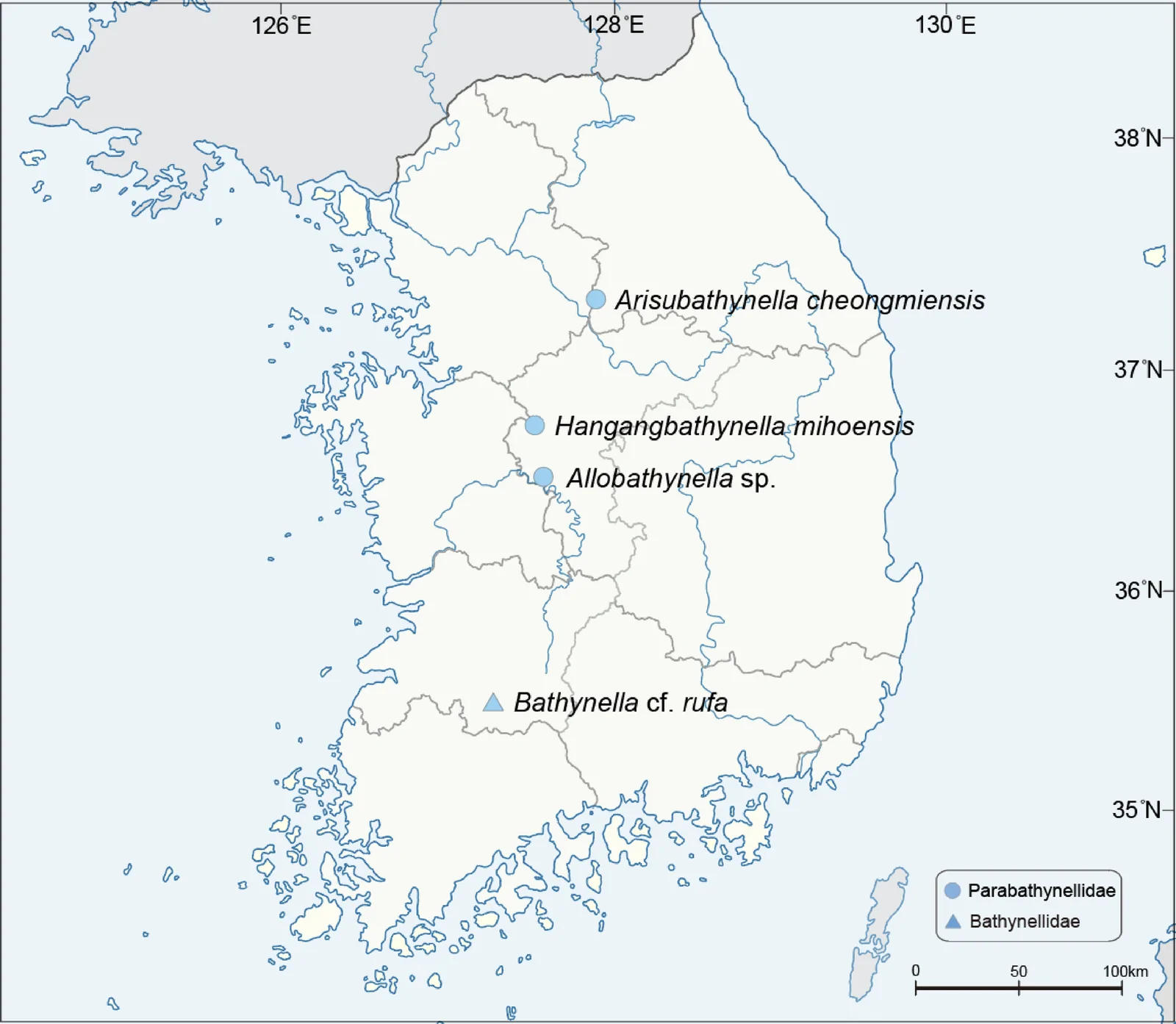
Collection sites of four bathynellacean species in South Korea.

Bathynellacean specimens were collected from interstitial hyporheic zones within aquatic subterranean habitats in South Korea (Fig. 6; Table 1) using a 1 m core and a manual pump, followed by filtration through a 50 μm mesh net^43^. Based on their morphological identification, we classified the specimens as *Allobathynella* sp., *Arisubathynella cheongmiensis*, *Hangangbathynella mihoensis*, and *Bathynella* cf. *rufa*, following the original descriptions^44–47^. Additionally, molecular identification was conducted based on *COX1* barcode sequences^46,48^.

### Mitochondrial DNA extraction, amplification, and sequencing

Mitochondria were isolated from live specimens using the Qproteome Mitochondria Isolation Kit (Qiagen, Hilden, Germany) following a 7-day starvation period at 8.0 °C in a biological oxygen demand incubator (VS-1203P5-L) to minimize potential contamination from food sources. Mitochondrial DNA was extracted using the LaboPass Tissue Genomic DNA Isolation Kit Mini (Cosmo Genetech, Seoul, South Korea). To compensate for the extremely low amount of DNA, we performed whole genome amplification using the REPLI-g Mitochondrial DNA Kit (Qiagen, Hilden, Germany) with Exonuclease-Resistant Random Primer 5′-NpNpNpNpNpSNpSN-3′ (Thermo Scientific, Hudson, NH, USA). To minimize amplification bias, three replicate whole genome amplification reactions were performed for each specimen. For mitogenome sequencing, 150-bp paired-end libraries were constructed using the Illumina TruSeq DNA Library Preparation Kit (Illumina, San Diego, CA, USA) and sequenced on the Illumina HiSeq X platform (Illumina, USA) at Macrogen (Seoul, Korea).

### Mitogenome assembly and annotation

Based on the *COX1* barcoding region, mitogenomes were assembled using a tool of “Map to Reference(s)” with Geneious Prime v.2024.0.2 (Biomatters Ltd., Auckland, New Zealand). Subsequently, the assembled mitogenome sequences were annotated using MITOS 2.1.7^49^ and refined manually using Geneious Prime v.2023.0.1 (Biomatters Ltd., New Zealand).

When we failed to recover complete mitogenomes, including a putative control region (CR) from *Allobathynella* sp. and *H. mihoensis*, using bioinformatics approaches, additional steps involving polymerase chain reaction (PCR) amplification, cloning, and Sanger sequencing were employed. Successful amplification was achieved only for the CR of *Allobathynella* sp. using manually designed primers (14939-F: 5′-ATGAACTTCTAACATATTAG-3′ and 235-R: 5′-TCGATAAGAAAGTTTACGAC-3′). The resulting PCR product was cloned using the T-Blunt™ PCR Cloning Kit (SolGent, Daejeon, Korea), and plasmid purification was conducted using the LaboPass™ Plasmid Mini Purification Kit (COSMOGENTECH, Seoul, Korea). Sanger sequencing was performed by Macrogen (Seoul, Korea) using universal primers (M13F and M13R-pUC) and additional internal primers.

### Mitogenome characterization

Genetic features, such as length, nucleotide composition, amino acid frequency, and relative synonymous codon usage, were determined using MEGA X^50^ and Geneious Prime v.2024.0.2 (Biomatters Ltd., Auckland, New Zealand). To assess global strand asymmetry, skew was calculated using the following formulae proposed by Perna and Kocher^51^: GC skew = (G − C)/(G + C) and AT skew = (A − T)/(A + T). To investigate structural elements within a CR, tandem repeat motifs were identified using the Tandem Repeats Finder program^52^, and their potential secondary structures were predicted using the RNAfold web server (http://rna.tbi.univie.ac.at//cgi-bin/RNAWebSuite/RNAfold.cgi). Transfer RNA (tRNA) secondary structures were predicted using MITOS 2.1.7^49^. Interspecific genetic divergence was calculated using the *p*-distance model implemented in MEGA X^50^.

For principal component analysis (PCA), the normality of amino acid frequency distributions across species was assessed using the Kolmogorov–Smirnov and Shapiro–Wilk normality tests; amino acids that were normally distributed (*p* > 0.05) were selected for analysis. The first two principal components (PC1 and PC2) were identified based on a scree plot, and the corresponding score plots were generated to explore potential patterns in amino acid composition among the Syncarida taxa. All statistical analyses were performed using R v4.4.1.

### Phylogenetic analyses

To reduce topological sensitivity to ingroup composition frequently observed in malacostracan phylogenetics, we constructed our dataset by appending newly obtained sequences to the framework established by Höpel et al.^53^. These selection criteria ensured broad taxonomic representativeness and helped minimize potential phylogenetic artifacts, while providing a stable backbone for our analysis (Supplementary material Table S1). Each mitochondrial protein-coding gene (PCG), excluding stop codons, was individually aligned, and all 13 PCGs were concatenated using Geneious Prime ver. 2024.0.2 (Biomatters Ltd., Auckland, New Zealand). Model selection for Bayesian inference (BI) was performed using jModelTest v2.1.10^54^, followed by identification of the best-fit substitution model based on the Akaike Information Criterion. The selected model was then applied to the BI analyses conducted using MrBayes v3.2.7^55^. Markov chain Monte Carlo runs were executed for 10 million generations, and the first 30% of the sampled trees were discarded as burn-in. The resulting phylogenetic trees were visualized and annotated using FigTree v1.4.4^56^. Maximum likelihood (ML) phylogenetic trees were constructed using IQ-TREE v1.6.8, with model selection performed under default settings and 1,000 ultrafast bootstrap replicates to assess nodal support^57^.

## Supplementary Information

Below is the link to the electronic supplementary material.

## Data Availability

Sequences are available at NCBI (KY310669, KY310670, KY310671, and PX991774).
