# Supplementary material for: Mitogenomic phylogeny of the aquatic subterranean Bathynellacea (Crustacea, Malacostraca) and implications for the monophyly of Syncarida
Source: Sci Rep. 2026 Jun 15;16:27586. doi: 10.1038/s41598-026-56722-z (PMC13538689; doi:10.1038/s41598-026-56722-z)
Supplement: Supplementary file 1 [file 41598_2026_56722_MOESM1_ESM.docx]

**Figure S1.** Predicted secondary structures of tRNAs in four Bathynellacea species (A–D).

**Figure S2.** Mitochondrial gene organization of Bathynellacea mitogenomes.

**Figure S3.** Phylogenetic trees based on the first and second codon positions of 13 mitochondrial protein coding genes from malacostracan species. Numbers at the nodes present posterior probabilities (left) and bootstrap values (right). Hyphens denote bootstrap values <50.

**Table S1.** GenBank accession numbers of mitogenomes used in this study.

| **Order** | **Species** | **Accession Number** |
| --- | --- | --- |
| **Bathynellacea** | ***Allobathynella* sp.** | **KY310669** |
| **Bathynellacea** | ***Arisubathynella cheongmiensis*** | **KY310670** |
| **Bathynellacea** | ***Hangangbathynella mihoensis*** | **XXXXXXXX** |
| **Bathynellacea** | ***Bathynella* cf. *rufa*** | **KY310671** |
| Amphipoda | *Acanthogammarus victorii* | KX341962 |
| Amphipoda | *Alicella gigantea* | MK215211 |
| Amphipoda | *Ampithoe lacertosa* | MK215645 |
| Amphipoda | *Brachyuropus grewingkii* | KP161875 |
| Amphipoda | *Caprella mutica* | GU130250 |
| Amphipoda | *Crangonyx forbesi* | MN175623 |
| Amphipoda | *Cyamus boopis* | MT458501 |
| Amphipoda | *Epimeria cornigera* | MF361127 |
| Amphipoda | *Eulimnogammarus cyaneus* | KX341964 |
| Amphipoda | *Eurythenes magellanicus* | MN688221 |
| Amphipoda | *Gammarus duebeni* | JN704067 |
| Amphipoda | *Garjajewia cabanisii* | KX341965 |
| Amphipoda | *Gmelinoides fasciatus* | KX341966 |
| Amphipoda | *Grandidierella taihuensis* | MW118119 |
| Amphipoda | *Haustorioides koreanus* | MN840593 |
| Amphipoda | *Hyalella azteca* | MH542433 |
| Amphipoda | *Jesogammarus hinumensis* | MT270126 |
| Amphipoda | *Metacrangonyx spinicaudatus* | HE860506 |
| Amphipoda | *Onisimus nanseni* | FJ555185 |
| Amphipoda | *Pallaseopsis kessleri* | KX341968 |
| Amphipoda | *Parhyale hawaiensis* | MH542432 |
| Amphipoda | *Platorchestia japonica* | MG010370 |
| Amphipoda | *Pleonexes koreana* | MK265245 |
| Amphipoda | *Pseudocrangonyx joolaei* | MT211951 |
| Amphipoda | *Pseudoniphargus carpalis* | MH592150 |
| Amphipoda | *Stygobromus allegheniensis* | MN175622 |
| Amphipoda | *Trinorchestia longiramus* | MH542431 |
| Anaspidacea | *Allanaspides helonomus* | OQ721879 |
| Anaspidacea | *Anaspides clarkei* | OQ721877 |
| Anaspidacea | *Anaspides tasmaniae* | OQ721893 |
| Anaspidacea | *Micraspides* sp. | OQ721895 |
| Cumacea | *Dimorphostylis asiatica* | MZ240751 |
| Decapoda | *Acetes chinensis* | JN689221 |
| Decapoda | *Alpheus japonicus* | MG787409 |
| Decapoda | *Alvinocaris chelys* | JX184903 |
| Decapoda | *Astacus astacus* | MT862440 |
| Decapoda | *Benthonectes filipes* | MF379624 |
| Decapoda | *Birgus latro* | KY352241 |
| Decapoda | *Caridina gracilipes* | KM023648 |
| Decapoda | *Cherax dispar* | HG799093 |
| Decapoda | *Dynomene pilumnoides* | KT182070 |
| Decapoda | *Engaeus lengana* | KF546209 |
| Decapoda | *Helicana wuana* | KX344898 |
| Decapoda | *Homarus americanus* | HQ402925 |
| Decapoda | *Lomis hirta* | KY352239 |
| Decapoda | *Maja crispata* | KY650651 |
| Decapoda | *Metapenaeus ensis* | MK500697 |
| Decapoda | *Neoglyphea inopinata* | KT984196 |
| Decapoda | *Orisarma sinense* | KR336554 |
| Decapoda | *Pagurus nigrofascia* | MH756635 |
| Decapoda | *Palaemon elegans* | MT340087 |
| Decapoda | *Panulirus japonicus* | AB071201 |
| Decapoda | *Paraglypturus tonganus* | KJ820739 |
| Decapoda | *Paratya australiensis* | KM978917 |
| Decapoda | *Penaeus japonicus* | AP006346 |
| Decapoda | *Penaeus monodon* | AF217843 |
| Decapoda | *Portunus pelagicus* | KM977882 |
| Decapoda | *Scyllarides latus* | KC107814 |
| Decapoda | *Typhlatya mitchelli* | KX844712 |
| Decapoda | *Upogebia major* | JN897377 |
| Euphausiacea | *Euphausia pacifica* | EU587005 |
| Euphausiacea | *Euphausia superba* | KX029326 |
| Euphausiacea | *Pseudeuphausia sinica* | MK579299 |
| Euphausiacea | *Thysanoessa raschii* | JN713150 |
| Isopoda | *Asotana magnifica* | MK790137 |
| Isopoda | *Bragasellus peltatus* | LR536607 |
| Isopoda | *Cymothoa indica* | MH396438 |
| Isopoda | *Eophreatoicus karrkkanj* | FJ790313 |
| Isopoda | *Gyge ovalis* | KY038053 |
| Isopoda | *Ichthyoxenos japonensis* | MF419233 |
| Isopoda | *Idotea baltica* | DQ442915 |
| Isopoda | *Isocladus armatus* | OK245257 |
| Isopoda | *Ligia oceanica* | DQ442914 |
| Isopoda | *Limnoria quadripunctata* | KF704000 |
| Isopoda | *Mongoloniscus sinensis* | MG709492 |
| Isopoda | *Proasellus ebrensis* | LR536613 |
| Isopoda | *Tachaea chinensis* | MF419232 |
| Mysida | *Neomysis awatschensis* | MN274520 |
| Mysida | *Neomysis japonica* | KR006340 |
| Stomatopoda | *Alima pacifica* | MW867307 |
| Stomatopoda | *Chorisquilla orientalis* | MT672286 |
| Stomatopoda | *Dictyosquilla foveolata* | MW864094 |
| Stomatopoda | *Faughnia haani* | MW632159 |
| Stomatopoda | *Gonodactylaceus randalli* | MW019425 |
| Stomatopoda | *Gonodactylellus affinis* | MW301115 |
| Stomatopoda | *Gonodactylus chiragra* | DQ191682 |
| Stomatopoda | *Harpiosquilla harpax* | AY699271 |
| Stomatopoda | *Hemisquilla californiensis* | MW867302 |
| Stomatopoda | *Lophosquilla costata* | MT276143 |
| Stomatopoda | *Lysiosquillina maculata* | DQ191683 |
| Stomatopoda | *Mesacturoides brevisquamatus* | MW867304 |
| Stomatopoda | *Neogonodactylus bredini* | MW867301 |
| Stomatopoda | *Odontodactylus havanensis* | MW867300 |
| Stomatopoda | *Oratosquilla oratoria* | GQ292769 |
| Stomatopoda | *Pseudosquilla ciliata* | AY947836 |
| Stomatopoda | *Squilla mantis* | AY639936 |
| Stomatopoda | *Squilloides leptosquilla* | KR095170 |
| Stomatopoda | *Taku spinosocarinatus* | MT672285 |
| Tanaidacea | *Arctotanais alascensis* | LC597489 |
| Balanomorpha(outgroup) | *Amphibalanus amphitrite* | KF588709 |

**Table S2.** Characteristics of tandem repeat regions in the control regions (CRs) of three bathynellacean mitogenomes.

| **Species**  **(CR length, bp)** | **Repeat unit length**  **(bp, AT content %)** | **Repeat no.** | **Repeat sequence (5’ to 3’)** |
| --- | --- | --- | --- |
| *Allobathynella* sp.  (2,064) | 119  (47.9) | 9 | GCTCTTATCCACTCTTCAAAAAAGGGCCCGTACCATAACTACCGGGCTAGGCCGAGTGATATAATAAAATTAATTAGCCTACTCTTGGCGCCCCTCCCCTTTCCCCAGAGGGGAGGGGG |
| *Arisubathynella cheongmiensis*  (224) | 5  (80.0) | 5 | TTTCA |
| *Bathynella* cf. *rufa*  (697) | 31  (90.3) | 21 | TTTTAAAAATACTACTAGTAAAATTAAAAAA |

**Table S3.** Summary of statistical results for principal component analysis based on relative amino acid composition of mitochondrial protein-coding genes in syncarid mitogenomes.

| **Amino acid frequency (%)** | | | | | | | | | | | | | | | | | | | | | | |
| --- | --- | --- | --- | --- | --- | --- | --- | --- | --- | --- | --- | --- | --- | --- | --- | --- | --- | --- | --- | --- | --- | --- |
| **Order** | **Species** | | **Ala** | **Arg** | **Asn** | **Asp** | **Cys** | **Gln** | **Glu** | **Gly** | **His** | **Ile** | **Leu** | **Lys** | **Met** | **Phe** | **Pro** | **Ser** | **Thr** | **Trp** | **Tyr** | **Val** |
| Bathynellacea | *Allobathynella* sp. | | 4.5 | 1.4 | 4.4 | 1.6 | 0.6 | 1.6 | 2.5 | 4.9 | 1.9 | 9.5 | 16.4 | 3.5 | 8.9 | 8.5 | 3.2 | 9.9 | 5.1 | 2.5 | 4.2 | 4.9 |
|  | *Arisubathynella cheongmiensis* | | 2.7 | 1.6 | 4.7 | 1.7 | 0.8 | 1.6 | 2.4 | 4.4 | 1.9 | 11.7 | 15.6 | 3.4 | 9.6 | 9.5 | 2.9 | 9.6 | 4.4 | 2.2 | 5.1 | 4.4 |
|  | *Hangangbathynella mihoensis* | | 2.7 | 1.4 | 6.2 | 1.6 | 0.7 | 1.6 | 2.3 | 4.4 | 1.8 | 11.9 | 15.4 | 3.3 | 9.7 | 10.1 | 2.6 | 8.8 | 3.9 | 2.3 | 5.6 | 4 |
|  | *Bathynella* cf. *rufa* | | 3 | 1.4 | 5.7 | 1.4 | 0.5 | 1.6 | 2.3 | 4.3 | 1.8 | 11.9 | 15.2 | 3.8 | 10.8 | 9.7 | 2.8 | 8.8 | 4.3 | 2.3 | 4.4 | 3.8 |
| Anaspidacea | *Allanaspides helonomus* | | 6 | 1.6 | 3.3 | 1.9 | 0.9 | 1.9 | 2.3 | 6.2 | 2.2 | 8.6 | 16.2 | 2.5 | 3.6 | 8.8 | 3.9 | 11.2 | 4.8 | 2.6 | 3.9 | 6.1 |
|  | *Allanaspides hickmani* | | 5.8 | 1.6 | 3.4 | 1.8 | 1 | 1.9 | 2.3 | 6 | 2.2 | 8.6 | 16.2 | 2.5 | 5.2 | 9.3 | 3.8 | 10.9 | 4.8 | 2.7 | 3.7 | 6.3 |
|  | *Anaspides clarkei* | | 5.5 | 1.6 | 3.7 | 2 | 1 | 1.9 | 2 | 6 | 2.2 | 8.8 | 15.7 | 2.6 | 5.2 | 10.2 | 3.8 | 10.4 | 4.8 | 2.8 | 3.8 | 6 |
|  | *Anaspides swaini* | | 5.9 | 1.6 | 3.7 | 2 | 1 | 1.9 | 2 | 6.1 | 2.2 | 8.8 | 15.7 | 2.5 | 4.8 | 10.4 | 3.7 | 10.8 | 4.6 | 2.8 | 3.6 | 5.8 |
|  | *Anaspides richardsoni* | | 5.7 | 1.6 | 3.7 | 2 | 1 | 2 | 2.1 | 6.2 | 2.1 | 8.7 | 15.6 | 2.4 | 5.2 | 10.3 | 3.7 | 10.5 | 4.6 | 2.8 | 3.6 | 6 |
|  | *Anaspides jarmani* | | 5.7 | 1.6 | 3.7 | 2 | 1 | 1.9 | 2.1 | 6 | 2.2 | 8.7 | 15.6 | 2.6 | 5.3 | 10.2 | 3.8 | 10.4 | 4.7 | 2.8 | 3.8 | 6 |
|  | *Anaspides eberhardi* | | 5.9 | 1.6 | 3.7 | 2 | 1 | 1.9 | 2.1 | 6.2 | 2.2 | 8.7 | 15.7 | 2.3 | 4.9 | 10.1 | 3.7 | 10.9 | 4.6 | 2.8 | 3.6 | 6.1 |
|  | *Anaspides spinulae* | | 5.7 | 1.6 | 3.6 | 2 | 0.9 | 1.9 | 2.1 | 6.3 | 2.1 | 8.6 | 15.6 | 2.4 | 5.1 | 10.2 | 3.7 | 10.7 | 4.7 | 2.7 | 3.7 | 6.2 |
|  | *Anaspides driesseni* | | 5.8 | 1.6 | 3.6 | 1.9 | 1 | 1.9 | 2.2 | 6 | 2.2 | 8.7 | 15.7 | 2.4 | 5.1 | 10.2 | 3.7 | 10.9 | 4.8 | 2.8 | 3.6 | 5.7 |
|  | *Anaspides tasmaniae* | | 5.6 | 1.6 | 3.7 | 2 | 1 | 1.9 | 2.1 | 6.1 | 2.2 | 8.8 | 15.7 | 2.4 | 5.3 | 10.4 | 3.7 | 10.9 | 4.5 | 2.8 | 3.7 | 5.7 |
|  | *Paranaspides lacustris* | | 5.8 | 1.6 | 3.7 | 2 | 1 | 1.9 | 2 | 6.4 | 2.3 | 7.8 | 15.6 | 2.5 | 5.5 | 9.7 | 3.8 | 10.3 | 4.8 | 2.8 | 3.8 | 6.7 |
|  | *Paranaspides williamsi* | | 5.9 | 1.6 | 3.6 | 2 | 1 | 1.9 | 2.1 | 6.2 | 2.3 | 8 | 15.9 | 2.4 | 5.4 | 9.7 | 3.9 | 10.4 | 4.6 | 2.7 | 3.7 | 6.6 |
|  | *Micraspides* sp. | | 4.7 | 1.5 | 4.2 | 1.8 | 1.1 | 1.7 | 2.4 | 5.8 | 2 | 9.8 | 15.8 | 2.7 | 5.9 | 9.6 | 3.6 | 10.4 | 4.9 | 2.8 | 4.1 | 5.3 |
| **Statistics** | | | | | | | | | | | | | | | | | | | | | | |
| Mean | | | 5.11 | 1.56 | 4.04 | 1.86 | 0.91 | 1.82 | 2.19 | 5.74 | 2.11 | 9.27 | 15.74 | 2.72 | 6.21 | 9.82 | 3.55 | 10.34 | 4.64 | 2.66 | 3.99 | 5.62 |
| Standard deviation | | | 1.18 | 0.08 | 0.8 | 0.19 | 0.17 | 0.14 | 0.16 | 0.73 | 0.16 | 1.3 | 0.3 | 0.47 | 2.11 | 0.55 | 0.41 | 0.7 | 0.29 | 0.21 | 0.57 | 0.86 |
| Coefficient of variation (%) | | | 23.1 | 5.13 | 19.8 | 10.22 | 18.68 | 7.69 | 7.31 | 12.72 | 7.58 | 14.02 | 1.91 | 17.28 | 33.98 | 5.6 | 11.55 | 6.77 | 6.25 | 7.89 | 14.29 | 15.3 |
| **Normality test** | | | | | | | | | | | | | | | | | | | | | | |
| Kolmogorov-Smirnov test | | Significance | 0.044 | 0.001 | 0.02 | 0.104 | 0.031 | 0.006 | 0.214 | 0.033 | 0.084 | 0.033 | 0.261 | 0.084 | 0.042 | 0.357 | 0.029 | 0.276 | 0.479 | 0.126 | 0.135 | 0.276 |
|  |  | Statistics | 0.335 | 0.462 | 0.367 | 0.295 | 0.35 | 0.414 | 0.256 | 0.348 | 0.305 | 0.347 | 0.196 | 0.305 | 0.337 | 0.225 | 0.353 | 0.241 | 0.204 | 0.285 | 0.281 | 0.241 |
| Shapiro-Wilk test | | Significance | 9.60E-05 | 3.39E-06 | 1.77E+00 | 6.90E-04 | 3.99E-04 | 9.05E-05 | 0.063 | 1.98E-04 | 0.007 | 4.13E-04 | 0.06 | 4.10E-04 | 4.73E-04 | 0.02 | 2.94E-04 | 0.008 | 0.079 | 2.00E-04 | 1.40E-04 | 0.026 |
|  |  | Statistics | 0.693 | 0.548 | 0.716 | 0.765 | 0.746 | 0.691 | 0.898 | 0.72 | 0.836 | 0.747 | 0.897 | 0.747 | 0.752 | 0.867 | 0.735 | 0.84 | 0.904 | 0.721 | 0.707 | 0.875 |

**Table S4.** Relative synonymous codon usage (RSCU) values at the third codon position of mitochondrial protein-coding genes in four bathynellacean mitogenomes (A–D).

**(A) *Allobathynella* sp.	(B) *Arisubathynella cheongmiensis***

| **Amino**  **Acid** | **RSCU at 3rd codon position** | | | |
| --- | --- | --- | --- | --- |
|  | **U** | **C** | **A** | **G** |
| Ala | 1.99 | 0.71 | 1.1 | 0.2 |
| Arg | 1.33 | 0.24 | 2.27 | 0.16 |
| Asn | 1.46 | 0.54 | - | - |
| Asp | 1.27 | 0.73 | - | - |
| Cys | 1.13 | 0.87 | - | - |
| Gln | - | - | 1.77 | 0.23 |
| Glu | - | - | 1.69 | 0.31 |
| Gly | 0.7 | 0.47 | 1.92 | 0.9 |
| His | 1.25 | 0.75 | - | - |
| Ile | 1.69 | 0.31 | - | - |
| Leu1 | 0.97 | 0.15 | 1.08 | 0.13 |
| Leu2 | - | - | 3.4 | 0.27 |
| Lys | - | - | 1.64 | 0.36 |
| Met | - | - | 1.79 | 0.21 |
| Phe | 1.76 | 0.24 | - | - |
| Pro | 1.95 | 0.56 | 1.43 | 0.07 |
| Ser1 | 1.02 | 0.55 | 2.41 | 0.6 |
| Ser2 | 1.97 | 0.35 | 0.99 | 0.11 |
| Thr | 1.95 | 0.56 | 1.41 | 0.09 |
| Trp | - | - | 1.64 | 0.36 |
| Tyr | 1.38 | 0.62 | - | - |
| Val | 1.72 | 0.56 | 1.51 | 0.2 |

| **Amino Acid** | **RSCU at 3rd codon position** | | | |
| --- | --- | --- | --- | --- |
|  | **U** | **C** | **A** | **G** |
| Ala | 2.63 | 0.16 | 1.17 | 0.04 |
| Arg | 2.21 | 0.07 | 1.43 | 0.29 |
| Asn | 1.67 | 0.33 | - | - |
| Asp | 1.75 | 0.25 | - | - |
| Cys | 1.42 | 0.58 | - | - |
| Gln | - | - | 1.69 | 0.31 |
| Glu | - | - | 1.73 | 0.27 |
| Gly | 1.3 | 0.35 | 2.05 | 0.3 |
| His | 1.68 | 0.32 | - | - |
| Ile | 1.87 | 0.13 | - | - |
| Leu1 | 0.7 | 0.01 | 0.76 | 0.06 |
| Leu2 | - | - | 4.04 | 0.43 |
| Lys | - | - | 1.66 | 0.34 |
| Met | - | - | 1.82 | 0.18 |
| Phe | 1.84 | 0.16 | - | - |
| Pro | 1.94 | 0.12 | 1.79 | 0.16 |
| Ser1 | 1.29 | 0.35 | 1.61 | 0.32 |
| Ser2 | 2.9 | 0.09 | 1.24 | 0.18 |
| Thr | 2.2 | 0.3 | 1.29 | 0.2 |
| Trp | - | - | 1.82 | 0.18 |
| Tyr | 1.8 | 0.2 | - | - |
| Val | 1.65 | 0.2 | 1.95 | 0.2 |

**(C) *Hangangbathynella mihoensis*	(D) *Bathynella* cf. *rufa***

| **Amino Acid** | **RSCU at 3rd codon position** | | | |
| --- | --- | --- | --- | --- |
|  | **U** | **C** | **A** | **G** |
| Ala | 2.43 | 0.15 | 1.35 | 0.07 |
| Arg | 1.33 | 0 | 2.59 | 0.08 |
| Asn | 1.82 | 0.18 | - | - |
| Asp | 1.72 | 0.28 | - | - |
| Cys | 2 | 0 | - | - |
| Gln | - | - | 1.86 | 0.14 |
| Glu | - | - | 1.86 | 0.14 |
| Gly | 1.38 | 0.08 | 2.21 | 0.33 |
| His | 1.88 | 0.12 | - | - |
| Ile | 1.95 | 0.05 | - | - |
| Leu1 | 0.41 | 0.02 | 0.22 | 0.02 |
| Leu2 | - | - | 5.24 | 0.09 |
| Lys | - | - | 1.93 | 0.07 |
| Met | - | - | 1.89 | 0.11 |
| Phe | 1.89 | 0.11 | - | - |
| Pro | 2.47 | 0.08 | 1.45 | 0 |
| Ser1 | 0.96 | 0.08 | 2.07 | 0.13 |
| Ser2 | 3.28 | 0.1 | 1.34 | 0.05 |
| Thr | 2.22 | 0.05 | 1.73 | 0 |
| Trp | - | - | 1.9 | 0.1 |
| Tyr | 1.87 | 0.13 | - | - |
| Val | 1.81 | 0.09 | 2.01 | 0.09 |

| **Amino Acid** | **RSCU at 3rd codon position** | | | |
| --- | --- | --- | --- | --- |
|  | **U** | **C** | **A** | **G** |
| Ala | 2.38 | 0.36 | 1.21 | 0.04 |
| Arg | 1.52 | 0.08 | 2.32 | 0.08 |
| Asn | 1.88 | 0.12 | - | - |
| Asp | 1.75 | 0.25 | - | - |
| Cys | 1.62 | 0.38 | - | - |
| Gln | - | - | 1.83 | 0.17 |
| Glu | - | - | 1.78 | 0.22 |
| Gly | 1.24 | 0.42 | 1.91 | 0.42 |
| His | 1.78 | 0.22 | - | - |
| Ile | 1.83 | 0.17 | - | - |
| Leu1 | 0.59 | 0.06 | 0.74 | 0.03 |
| Leu2 | - | - | 4.28 | 0.3 |
| Lys | - | - | 1.77 | 0.23 |
| Met | - | - | 1.88 | 0.12 |
| Phe | 1.84 | 0.16 | - | - |
| Pro | 2.45 | 0.13 | 1.33 | 0.09 |
| Ser1 | 1.4 | 0.13 | 2.28 | 0.3 |
| Ser2 | 2.63 | 0.28 | 0.93 | 0.08 |
| Thr | 2.35 | 0.09 | 1.53 | 0.03 |
| Trp | - | - | 1.81 | 0.19 |
| Tyr | 1.84 | 0.16 | - | - |
| Val | 1.85 | 0.11 | 1.82 | 0.22 |
